# Neuromyelitis Optica Spectrum Disorder: From Basic Research to Clinical Perspectives

**DOI:** 10.3390/ijms23147908

**Published:** 2022-07-18

**Authors:** Tzu-Lun Huang, Jia-Kang Wang, Pei-Yao Chang, Yung-Ray Hsu, Cheng-Hung Lin, Kung-Hung Lin, Rong-Kung Tsai

**Affiliations:** 1 Department of Ophthalmology, Far Eastern Memorial Hospital, Banqiao Dist., New Taipei City 220, Taiwan; jiakangw2158@gmail.com (J.-K.W.); peiyao@seed.net.tw (P.-Y.C.); scherzoray@gmail.com (Y.-R.H.); 2 Department of Electrical Engineering, Yuan Ze University, Chung-Li, Taoyuan 320, Taiwan; chlin@saturn.yzu.edu.tw; 3 Department of Medicine, National Yang-Ming University, Taipei City 112, Taiwan; 4 Department of Medicine, National Taiwan University, Taipei City 106, Taiwan; 5 Department of Healthcare Administration and Department of Nursing, Oriental Institute of Technology, New Taipei City 220, Taiwan; 6 Biomedical Engineering Research Center, Yuan Ze University, Taoyuan 320, Taiwan; 7 Department of Neurology, Taiwan Adventist Hospital, Taipei City 105, Taiwan; kksao.lin@gmail.com; 8 Institute of Eye Research, Hualien Tzu Chi Hospital, Buddhist Tzu Chi Medical Foundation, Tzu Chi University, 707 Sec. 3 Chung-Yung Road, Hualien 970, Taiwan; 9 Institute of Medical Sciences, Tzu Chi University, Hualien 970, Taiwan

**Keywords:** neuromyelitis optica spectrum disease, aquaporin-4, myelin oligodendrocyte glycoprotein, ocular coherence tomography, complement, microcystic macular degeneration, Müller cell, astrocyte, oligodendrocyte, microglia

## Abstract

Neuromyelitis optica spectrum disorder (NMOSD) is an inflammatory disease of the central nervous system characterized by relapses and autoimmunity caused by antibodies against the astrocyte water channel protein aquaporin-4. Over the past decade, there have been significant advances in the biologic knowledge of NMOSD, which resulted in the IDENTIFICATION of variable disease phenotypes, biomarkers, and complex inflammatory cascades involved in disease pathogenesis. Ongoing clinical trials are looking at new treatments targeting NMOSD relapses. This review aims to provide an update on recent studies regarding issues related to NMOSD, including the pathophysiology of the disease, the potential use of serum and cerebrospinal fluid cytokines as disease biomarkers, the clinical utilization of ocular coherence tomography, and the comparison of different animal models of NMOSD.

## 1. Introduction

Neuromyelitis optica spectrum disorder (NMOSD) is a common cause of optic neuritis (ON) in Taiwan. In 2015, the prevalence of NMOSD was 1.47/100,000, and the age-standardized annual incidence rate was 0.61/100,000 person-years [1]. The reported prevalence of NMOSD in different racial groups is approximately 1/100,000 in White individuals, 3.5/100,000 in Asians, and 10/100,000 in Black individuals [2]. The differential diagnosis of NMOSD and multiple sclerosis (MS) was challenging until the discovery of neuromyelitis optica (NMO) autoantibodies by Lennon et al. [3,4]. In most cases, NMOSD is caused by pathogenic NMO immunoglobulin G (IgG) autoantibodies that bind to the aquaporin-4 (AQP4) target antigen, a water channel expressed on the end-feet membranes of astrocytes along the blood–brain barrier (BBB) and in Müller cells distributed on the fovea centralis in the retina [4,5,6,7,8,9]. The pathology most often occurs in the periventricular zone, involving astrocyte plasma membrane domains facing the pia and vessels, whereas the least-affected site in the central nervous system (CNS) is the area postrema in the dorsal medulla [10,11]. 

Currently, the clinical diagnosis of NMOSD is mainly based on the detection of serum NMO-IgG (AQP4-IgG) antibodies and the presence of core symptoms included in the diagnostic criteria developed by the International Panel for NMO Diagnosis in 2015 (Table 1) [10,12,13]. The revised criteria that replaced the previous 2006 criteria for NMO diagnosis resulted in a significant increase in the diagnostic sensitivity of NMOSD by 76% (62% in the AQP4-IgG-positive group and 14% in the seronegative group) [14]. For AQP4-IgG-positive patients, at least one of six sites within the CNS, including the spinal cord, optic nerves, area postrema, diencephalon, brainstem, and cerebrum, must be attacked. In seronegative patients, at least two core sites have to be affected and additional magnetic resonance imaging (MRI) criteria fulfilled [13]. The rate of seropositivity for myelin oligodendrocyte glycoprotein (MOG-IgG) antibodies in AQP4-IgG-seronegative patients with NMOSD was reported to reach up to 41.6% [15].

From the perspective of clinical application, biological biomarkers may be important for predicting the future risk of relapse and disease prognosis [10,16]. AQP4-IgG antibody titers seem to be linked to clinical presentation and immune response, with higher titers associated with worse visual function and more extensive cerebral involvement on MRI [16]. On the other hand, AQP4-IgG antibodies might represent a byproduct resulting from complex immunoinflammatory processes in NMOSD, with no significant variations in antibody titers between different disease stages [17]. Beyond autoantibodies, the clinical presentation and demographic features may be more reliable in terms of prognosis prediction [18]. Age was reported to be predictive of the involvement site, and ON seems to be the most common inflammatory lesion in NMOSD patients younger than 30 years [19].

AQP4-IgG-seropositive NMOSD indicates the entity of astrocytopathy, and MOG-IgG is a protein expressed by oligodendrocytes on the most superficial surface of myelin sheaths, which results in oligodendropathy [20,21,22]. MOG antibody-associated disease (MOGAD) exhibits different clinical features from those of AQP4-IgG-seropositive NMOSD [23,24,25]. The phenotype difference between AQP4-IgG- and MOG-IgG-positive ON can be assessed by the length of the optic nerve involvement and preferable involvement site on MRI, the morphology of the optic disc, laterality, and the pattern of the ganglion cell–inner plexiform layer (GC-IPL) on optical coherence tomography (OCT) [24,26]. MRI image characteristics add evidence to the differential diagnosis of ON. AQP4-ON preferentially presents with a longer, more unilateral, more posterior portion of the optic nerve with T1 gadolinium enhancement [13,27,28]. In contrast, MOG-ON usually presents with a longer, more bilateral and more anterior portion of the optic nerve accompanied by intraorbital optic nerve swelling and perineural T1 gadolinium enhancement [29].

Sex difference is low due to the higher proportion of males in the MOGAD group compared with that in the AQP4-IgG group. For MOGAD, 41% (7/17 case) to 44% (4/9 case) of female cases were noted [24,30]. MOG-ON may present at multiple ages and shows no sex bias, but the female/male ratio is 7.2:1.0 in the AQP4-ON group [26,29,31]. Moreover, only 9% of MOG-IgG-positive cases have a concurrent autoimmune disorder, and 80% of MOGAD cases will relapse but have a better clinical prognosis [32,33].

Although most neuronal damage occurs during the first episode, the treatment of a relapse episode in patients with NMOSD is essential for preserving as much of the neuron reservoir as possible. Most patients with NMOSD achieve good functional improvement after corticosteroid treatment and add-on plasmapheresis in the acute stage. However, clinical relapse occurs in most cases, resulting in cumulative neurologic damage. The new strategies may provide additional options for patients who are refractory to current maintenance therapies including treatments interfering with eosinophilic function, monoclonal antibodies that target neutrophil elastase, complement activation, interleukin IL-6 receptor (IL-6R) signaling, and plasma cells producing AQP4, and MOG-IgG antibodies [34,35,36,37,38,39,40]. Recently, three monoclonal antibody therapies approved by the Food and Drug Administration for the treatment of NMOSD demonstrated safety and efficacy in reducing the risk of relapse during remission; these are eculizumab (inhibitor of complement protein C5), inebilizumab (humanized monoclonal antibody against CD19 B cell protein), and satralizumab (humanized recombinant monoclonal antibody targeting IL-6R).

## 2. The Pathogenesis of NMOSD

AQP4 contributes to the stabilization of extracellular osmolality during neuronal activity. Moreover, it maintains glutamate homeostasis and energy balance as well as buffers the metabolic load in the BBB [5,41]. The pathological features of NMOSD include activated complement with extensive vasculocentric immune complex deposition, the loss of AQP4 expression in astrocytes, neutrophil/macrophage/microglial infiltration and eosinophil degranulation, myelin loss, and thickened hyalinization blood [4,5,42,43,44,45].The two major AQP4 isoforms, M1 and M23, exhibit locational and maturational differences in the ratio of M1 to M23 proteins along the astrocytic membrane, which possibly determines the pathogenicity and a different anatomical distribution in the CNS and at different stages of CNS maturation in pediatric and adult patients [46,47,48,49]. The proportion of the largest AQP4 aggregate is the highest in the optic nerve followed by the spinal cord; this is relevant to why NMO selectively targets the CNS tissue and spares non-CNS AQP4-expressing tissues [50]. The M1 protein is completely internalized, but M23 resists internalization and activates the complement more efficiently than M1 when bound by the antigen [46,51]. The relative components of AQP4 isoforms are tissue-specific, with an approximate 3:1 ratio of AQP4-M23 to AQP4-M1 in rat brain [52]. Formation of supramolecular structures, called orthogonal arrays of particles (OAPs), by AQP4 is essential in NMOSD pathogenesis and enhances complement-dependent cytotoxicity (CDC) by the pathogenic AQP4-IgG [53]. It remains unclear if the OAP composition varies in pediatric and adult patients or whether OAP differences may cause different phenotypes [54].

Müller cells and neuronal axons are the main targets in an experimental model of NMO [55]. After intravitreal injection of AQP4-IgG antibodies, complement activation and immunoglobulin deposition was found in Müller cells and caused a retinal pathology [56]. AQP4 is also coexpressed with the Kir4.1 potassium channel subunit in cells, and the electrogenic bicarbonate transporter contributes to changes in the extracellular space, involved in buffering K+ [8]. In clinical practice, Müller cell dysfunction was shown to significantly reduce the b-wave amplitude in the scotopic electroretinogram of AQP4-IgG-positive patients compared with normal controls [57]. However, the results were inconclusive regarding a relationship between the b-wave amplitude and the volume of the outer retinal segment on OCT as well as disease severity, assessed on the basis of the Expanded Disability Status Scale (EDSS) or visual acuity [57].

## 3. Cytotoxicity Pathway

Complement-dependent cytotoxicity (CDC), complement-dependent cellular cytotoxicity (CDCC), and antibody-dependent cellular cytotoxicity (ADCC) are responsible for astrocyte injury in NMOSD [58,59]. ADCC seems to play a main role in facilitating macrophage and natural killer (NK) cell activation after binding to the CH3 region of IgG antibodies via the effector cells’ Fc receptors in the outer zone of developing lesions (penumbra) [59,60,61]. In CDC, antibody binding to a target antigen triggers the classic complement pathway and results in the formation of the membrane attack complex (MAC). In CDCC, another protein, C3b, is expressed during the complement cascade activation and interacts with NK cells and macrophages to facilitate cell lysis [62].

**Figure 1:**
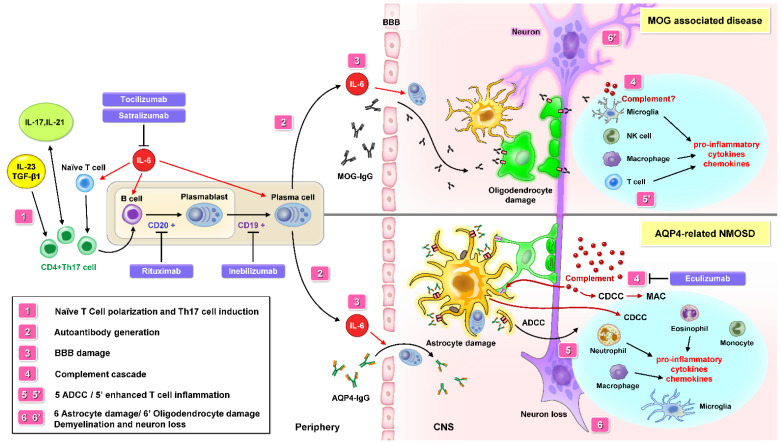
The pathological mechanism of NMOSD may involve peripheral autoimmune dysregulation. Interleukin 6 (IL-6) is a key factor in AQP4-related NMOSD pathophysiology. A similar role of IL-6 is also reported in MOG-associated disease (MOGAD). Besides IL-6, Th17 cells differentiation may be induced by IL-17, IL-21, IL-23, and TGF-β1. It is thought that an impaired innate immune system may promote naive T cell transformation into Th 17 cell and stimulate B cell differentiation to plasmablasts, then to plasma cells producing AQP4-IgG or MOG-IgG autoantibody. A leaky BBB contributes to the migration of AQP4-IgG from the periphery into the CNS. AQP4-IgG bind to AQP4 and activate the complement cascade (CDC and CDCC) and ADCC. Cytokine and chemokine production leads to the recruitment of macrophages, eosinophils, neutrophils, and monocytes to the inflammation site. After microglia and macrophage infiltration, astrocytes and oligodendrocyte are damaged, which leads to advanced axonal degeneration and neuronal death. AQP4-IgG-seropositivity in NMOSD indicates the entity of astrocytopathy, and MOG-IgG results in oligodendropathy, named MOGAD. Current maintenance therapies include interfering with complement activation (Eculizumab), IL-6 R signaling (Tocilizumab and Satralizumab), and plasma cells producing AQP-4 and MOG IgG Abs (Rituximab and Inebilizumab). Abbreviations: NMOSD = neuromyelitis optica spectrum disorders; BBB = blood–brain barrier; CDC = complement-dependent cytotoxicity; CDCC = complement-dependent cellular cytotoxicity; ADCC = antibody-dependent cellular cytotoxicity; MOG = myelin oligodendrocyte glycoprotein; AQP-4 = aquaporin-4; IL-6 = interleukin 6; IL-17 = interleukin 17; Th17 cell = T helper 17 cell; TGF-β1 = transforming growth factor beta 1; IL-6 R = IL-6 receptor.

Peripheral autoimmune dysregulation starts after the modulation of peripheral T cells. Pathogenic autoreactive T cells (Th17 cells) and IL-6 disrupt BBB tight junctions, resulting in CNS inflammation due to the effect of numerous chemokines and cytokines (Figure 1) [63,64,65]. IL-6 is a proinflammatory cytokine that amplifies inflammation, increases the survival of plasmablasts capable of producing AQP4-IgG antibodies, supports the differentiation of B cells to plasma cells, and induces BBB injury [66,67]. Because B cells and autoantibodies were found to be disease beginners in experimental autoimmune encephalomyelitis (EAE), inebilizumab is instructive for modeling the therapeutic effects with enhanced ADCC against CD19-positive B cells, as confirmed in MS and NMOSD [68,69].

The activation of the complement cascade in patients with NMOSD was reported to increase membrane permeability and promote the influx of serum AQP4-IgG antibodies, which further amplified the inflammatory reaction at the BBB of the CNS [70]. Basic research demonstrated that AQP4 antibodies trigger the complement system and lead to MAC formation via the CDC pathway, which results in astrocyte damage and secondary neuronal injury [71,72]. C1q-targeted monoclonal antibodies were demonstrated to effectively inhibit AQP4-IgG-mediated CDC, which interfered with MAC, and also IgG-mediated CDCC, which influenced the formation of the Cb3–Cb3R complex on macrophage and NK cells in an in vivo study [73]. Eculizumab is a humanized monoclonal antibody that inhibits terminal C5 complement protein cleavage into the C5a (inducing proinflammatory activity) and C5b fragments (inducing the MAC formation) [74,75,76]. Serum C4 levels were found to be lower in patients with AQP4-IgG-positive NMOSD in clinical remission than in those with MOGAD and MS as well as in healthy controls [77]. Immune features and the cytokine profile in the cerebrospinal fluid (CSF) significantly vary in patients with MS, AQP4-positive NMOSD, and MOGAD, suggesting that these are different autoimmune demyelinating diseases [78,79]. The role of complement in MOGAD has not been fully elucidated so far. It is possible that MOG-IgG could cause reversible myelin damage without complement activation [80]. On the other hand, a subset of human MOG-IgG antibodies was shown to induce complement-dependent pathogenic effects in a murine animal model [81]. Increased levels of proteins indicating classic and alternative complement activation were observed in patients with MOGAD compared with the control groups. Therefore, complement activation could be a potential therapeutic target in patients with MOGAD [82].

The new evidence on NMOSD pathophysiology highlights promising treatment modalities as well as clinical studies [83]. Restoring immune tolerance might provide an interesting treatment strategy in the future [84]. Some success was achieved by using autologous hematopoietic stem cell transplantation [85], peptide-loaded tolerogenic dendritic cells [86], DNA vaccine encoding myelin basic protein, [87], autoreactive T cell vaccination, and regulatory T cells [88,89]. Further alternative targets for NMOSD treatments are the blood–brain barrier, [90], the complement cascade [91], and B cells [92].

## 4. Genetic Susceptibility to NMOSD

Despite important breakthroughs in the understanding of AQP4 and MOG antibodies and their involvement in NMOSD, the genetic factors underlying the disease pathogenesis have not been fully understood. More recently, genome-wide single-nucleotide polymorphism arrays have shown some susceptibility loci for NMOSD [93]. It is predominantly a sporadic disorder, although familial NMOSD occurs in 3% of the cases [93,94]. Human leukocyte antigen (HLA) haplotypes were reported to be highly correlated with NMOSD. HLA is located on chromosome 6, and the main variations are observed in DQA1, DQB1, DRB1, and DPB1 [95,96,97]. Whole-genome sequence studies that have been conducted in Europeans since 2009 identified a C4A deletion and a fourfold reduction of C4a levels as the most likely functional drivers of an increased risk for AQP4-IgG production. Furthermore, HLA-DQA1*102, HLA-DQA1*501, HLA-DQB1*0201, and HLA-DRB1*03 alleles were significantly associated with NMOSD [96,98]. In Japan, Ogawa et al. found that HLA-DQA1*05:03 was significantly associated with NMOSD, whereas Watanabe et al. reported that HLA-DRB1*08:02 and HLA-DPB1*05:01 were associated with susceptibility to NMOSD and that HLA-DRB1*09:01 was protective against NMOSD [99,100,101]. In addition, distinct genetic and infectious profiles in Japanese patients with NMOSD demonstrated that the HLA-DRB1*1602 and HLA-DPB1*0501 alleles as well as infection with *Helicobacter* *pylori* and *Chlamydia pneumonia* were associated with higher susceptibility to AQP4-IgG-seropositive NMOSD [102]. Future studies should evaluate response to treatment as well as genetic and cytokine profiles in association with distinct genetic backgrounds in patients with NMOSD.

In summary, the potential molecular mechanisms underlying AQP4-seropositive NMOSD may be related to proteins encoded by the novel genes involved in complement activation, antigen presentation, antibody-dependent cytotoxicity, and immune regulation [103].

## 5. Potential Biomarkers in NMOSD

### 5.1. Surrogate Serum Biomarkers

Evidence shows that AQP-4 IgG antibodies are not strongly associated with clinical indices, such as the EDSS, risk of relapse, or visual prognosis in NMOSD [17]. On the other hand, there are data suggesting that the activation of complements, cytokines, and chemokines contributes to the complex pathogenesis of the disorder [104,105]. Naive T-helper cells differentiate into a new lineage called Th17 and have the capacity to produce large amounts of IL-17, a cytokine linked to autoimmune diseases [67,106]. IL-6 signaling involving Th17 cells and Th17-associated cytokines may play a crucial role in the pathogenesis of NMOSD (Figure 1) [66,107,108,109,110]. Apart from IL-17, Th17-cell differentiation may be induced by IL-6, IL-23, and transforming growth factor β1 [111,112]. Granulocyte macrophage colony-stimulating factor acts as a proinflammatory cytokine and could critically be involved in the formation of Th17 cells and the activation of macrophages and dendritic cells involving the secretion IL-23 and IL-6 [113]. Patients with NMOSD were shown to have higher levels of IL-6, IL-17, and IL-21 in both CSF and serum, as well as higher levels of IL-1, IL-8, IL-13, and granulocyte colony-stimulating factor in the CSF than those with MS [66,107]. The IL-6 levels in CSF correlate with neural damage biomarkers in NMOSD, and increased plasma IL-6 levels correlate with the EDSS [114]. As for the pathological mechanisms, IL6 signaling is thought to contribute in multiple ways, as shown in Figure 1. Two monoclonal antibodies, satralizumab and tocilizumab, activate the same mechanism, in that they both target IL-6 receptor- (IL-6R) and IL-6-associated immune cascades, leading to T-cell activation, IgG secretion, BBB damage, activation of the complement cascade, and enhancement of macrophage and microglia activity [84,109,115,116]. In a novel in vitro BBB model, the proposed role of IL-6 on the BBB was confirmed [117]. AQP4-IgG induced IL-6 release from astrocytes, then the BBB was impaired by IL-6 signaling in endothelial cells, and reversal of the BBB impairment was enhanced by anti-IL-6 receptor (IL-6R) antibodies [110,118,119].

Complex processes involving activated microglia ultimately promote the pathological course of NMOSD, and that suggests that microglia may serve as a therapeutic target in NMO [120]. Briefly, complement C3a secreted from activated astrocytes may induce the secretion of complement C1q and inflammatory cytokines by microglia, facilitating injury to microglia, astrocytes, oligodendrocytes, and neurons in an autocrine or paracrine manner [121].

Serum biomarkers including glial fibrillary acidic protein (GFAP) and neurofilament light chain (NfL) may help guide the design of effective therapies for the management of disease [22,26,122,123,124]. In a subgroup analysis, the CSF levels of IL-6, NfL, and GFAP were higher in AQP4-IgG-positive cases and might be used as indicators of disease activity, relapse risk, and therapy efficacy [124,125,126]. Factors involving the tight junctions seem to be other candidates for key biomarkers. Epidermal growth factor may be involved in the disruption of the BBB by downregulating claudin-5 in NMOSD, and women were shown to exhibit higher urinary levels of this factors, which might explain their greater susceptibility to NMOSD [127]. Interferon-γ reduces BBB integrity in cultured brain endothelial cells through Rho kinase-mediated cytoskeletal contraction, causing junction irregularity and cell–cell disconnections leading to deformity in adherence and tight junction proteins [128,129]. Serum vascular endothelial growth factor, myeloid progenitor inhibitory factor 1, and neuron–glia-related cell-adhesion molecule were positively associated with AQP4-IgG titers; thus, they could be potential biomarkers of NMOSD [127]. A study on plasma chemokine levels in NMOSD during remission confirmed that IL-1β and tumor necrosis factor α stimulate eosinophilic chemoattraction, suggesting that the elevated secretion of monocyte chemotactic protein (C–C motif chemokine ligand 13, CCL13) and eotaxins (CCL11 and CCL26) may be a critical step in eosinophil recruitment during remission [130].

### 5.2. OCT Biomarker

The international CROCTINO program uses OCT as a standardized method to assess the clinical course and pathophysiology of NMOSD as well as to monitor therapeutic efficacy [131,132]. OCT was shown to provide unique insights into the identification of foveal pitting in NMOSD likely due to the loss of Müller cells [133].

## 6. Optic Nerve Structure in NMOSD

The use of OCT to discriminate the microstructures of the retinal nerve fiber layer and GC-IPL has been debated in recent studies on NMOSD and MS. Moreover, while current therapies were demonstrated to improve the visual function after acute treatment, structural improvement remains an unmet need. GC-IPL thickness associated with visual ability in NMOSD-ON eyes leads to more severe retinal thinning and visual impairment than that found in MS [134]. A cross-sectional collaborative retrospective study reported that the average GC-IPL loss was 22.7 μm after the first ON attack, and the average loss after a recurrent episode was 3.5 μm, with noticeable subclinical GC-IPL loss in non-optic neuritis (NON) eyes [132]. NMOSD-NON eyes exhibited reduced thickness in the GC-IPL but not in the pRNFL compared with normal eyes, and relative changes in the parvocellular layer of NMOSD-NON eyes were not fully confirmed in recent studies [132,134,135]. Due to the presence of numerous confounding factors when determining the thickness of the anterior visual pathway, parafoveal segmentation on OCT may enable a more sensitive detection of neuronal loss and reflect a neurodegenerative reaction of retinal ganglion cells (RGC) and Müller cell damage in NMOSD [136,137]. Contrarily, pRNFL in the papillomacular bundle exhibited a reduction in MS-NON due to parvocellular axons being more vulnerable to energy depletion in MS studies [138,139,140]. GC-IPL damage on OCT spatial relationship and papillomacular bundle loss in neurodegenerative disease such as mitochondriopathy or neuroinflammmatory disease require further research to elucidate the damage in the megacellular and parvocellular layers in the lateral genicular nucleus [139,141]. There were no significant differences in the annual changes in mGC-IPL, pRNFL, total macular volume, and disc cup ratio in the NMOSD-ON eyes when comparing patients treated with different immunosuppressive therapies [142].

## 7. Macular Structure in NMOSD

The foveal structure on OCT has been discussed as a potential biomarker of NMOSD. The foveal structure, including foveal pit shape, depth or total macular volume (mm^3^), or central foveal thickness, may be an early diagnostic marker of NMOSD [136,143,144]. This hypothesis is supported by data from animal studies, which showed Müller cell death after intravitreal injection of AQP4 antibodies and revealed a lysosomal degradation mechanism for AQP4 loss on Müller cells [145].

In the parafoveal region, which is rich in astrocytes and AQP4-expressing Müller cells, a positive association between attack-independent neural loss and visual function was observed [57]. Microcystic macular degeneration (MMD) may contribute to loss in both high- and low-contrast visual testing after an NMOSD-ON episode [136,137]. The inner nuclear layer (INL) and outer retinal layer were thicker in the NMOSD-ON eyes compared with NMOSD-NON and control healthy eyes, due to the presence of MMD in the episode of ON [134,137,146]. MMD is predominantly localized in the INL, but also extends to the outer nuclear layer [136]. Interestingly, a previous study demonstrated that the INL thickness was negatively correlated with the GC-IPL content in NMOSD [147].

To date, there have been no cross-sectional and longitudinal studies to elucidate whether MMD is a temporary change or secondary to optic neuropathy. Although the pathophysiologic mechanism of MMD in NMO is complex, degeneration is believed to be caused by the disruption of the blood–retinal barrier and the transition of microglial cells to phagocytose apoptotic RGCs [148]. Another possibility is vitreomacular traction, but fluorescein angiography is needed to confirm the cause–effect relationship [149]. MMD exhibited no progression over a 20-month follow-up, and the risk of MMD was observed in 4.7% of patients with MS and in 13.3–26% of patients with NMOSD [136,137,150]. MMD is not a specific sign of NMOSD, and the exclusion of other secondary insults, such as uveitis, diabetes, or Fingolimod exposure, is required [150,151]. In hereditary optic neuropathy, MMD may be associated with vitreomacular traction or the epiphenomenon of optic atrophy, unrelated to inflammation or retrograde transsynaptic degeneration [152]. In advanced glaucomatous optic atrophy, MMD in the superior and nasal macular quadrants was also documented on OCT [147]. Furthermore, the INL cyst secondary to retinitis pigmentosa was not an uncommon sign [153,154].

To summarize, INL cystoid lesions are a nonspecific indicator of degenerative optic neuropathy or retinopathy, and MMD could be linked to Müller cell pathology in NMOSD [155,156,157]. However, further research is required to prove this hypothesis.

## 8. OCT Angiography in NMOSD

Subclinical vascular changes in the parafoveal retina might occur during an ON attack and could be associated with astrocyte damage with increased levels of sNfL/sGFAP [158]. Patients with NMOSD exhibited an enlargement of the foveal avascular zone independent of an ON attack [158]. This could be explained by damage of the blood–retinal barrier resulting from Müller cell loss, leading to the enlargement of the foveal avascular zone on OCT angiography in patients with NMOSD but not in those with MS [137,144,158,159]. A strong correlation between the deep vascular complex and visual acuity was reported, and decreased microvascular density in the superficial and deep vascular complex was significantly correlated with the frequency of NMO-ON attacks [160]. OCT angiography with a measurable analysis offers a new possibilities in the study of microvascular impairment in NMOSD and may become an objective clinical tool for patient monitoring.

## 9. Animal Models of Neuromyelitis Optica

The available animal models of NMO are largely based on a passive transfer of AQP4-IgG antibodies or AQP4-sensitized T cells to rodents and are often combined with proinflammatory maneuvers (coinfusion of proinflammatory factors or additional needle trauma) [60,161,162,163]. The models exhibit T cell and B cell activation, macrophage/microglial infiltration, eosinophil aggregation, immune complex deposition, loss of AQP4 and GFAP expression, and astrocyte/axonal injury [42,45,60,162] (Table 2). There are currently two main methods for generating animal models of NMO: one is NMO-IgG immunization in the EAE model [162,163,164,165,166], and the other is coinjection of the NMO-IgG/human complement into the target, which can be a route of intraventricular, intra-spinal cord, perichiasmal, or transoptic nerve sheath [42,92,161,167,168,169,170].

## 10. Animal Model of NMO

Passive immunization by intraperitoneal injection of AQP4-specific T cells in EAE rats allows EAE to develop faster via specific targeting of the astrocytes and the entry of pathogenic AQP4 antibodies to produce NMO-like lesions in the CNS after 10 to 14 days from the injection [171]. The extent and location of inflammation and demyelination mainly vary according to the specific antigen introduced and rodent species and strain [170]. Lewis rats can produce high titers of antibodies against specific epitopes of human AQP4 [172]. However, Saadoun et al. recently demonstrated that the coinjection of NMO-IgG with human complement could produce NMO-like lesions in naive mice without EAE [92]. Asavapanumas et al. reported that a single intracerebral or intraperitoneal injection of NMO-IgG antibodies after intracerebral needle stab injury without the administration of complement or proinflammatory cytokines was able to produce a mouse model of NMO [60,161]. Evidence suggests that the coadministration of the complement is required for developing a complete NMO lesion but is not needed for the development of the penumbra, which emphasizes the pathogenic role of macrophage/microglia involving ADCC in NMO [59,60].

Studies demonstrated that a passive transfer by intravenous or intraperitoneal injection of the AQP4 antibodies from seropositive NMO patients was insufficient to cause NMO-like lesions in rodents, as the low levels of antibodies could not penetrate into the CNS [163,173]. When applying patient sera without any filtration modification, it is likely that coexisting human complements may play a synergic role in the development of strong neuroinflammation [169,174,175]. Optic nerve susceptibility in NMO might also arise from the abundant AQP4 expression along the optic nerve compared with the brain [65]. Aside from the optic nerve, AQP4 is also highly expressed in astrocyte-like Müller cells in the inner retina and the ciliary epithelium [7]. A study using intravitreal AQP4-IgG passive transfer resulted in a complement-independent retinal pathology that reduced AQP4 expression and increased GFAP levels by 5 days [145]. We summarize the rodent models regarding ON without existence of EAE in Table 2. The passive cotransfer of NMO-IgG antibodies and complement via a continuous 3-day infusion near the optic chiasm in mice seemed to be sufficient to develop NMO-ON [42]. However, continuous infusion with precise needle placement is technically challenging and might cause additional irreversible damage. On the other hand, retrobulbar infusion, intravitreal injection, or a single intracranial injection may result in a limited or transient pathology [42]. We believe that local retrobulbar injection of NMO-IgG-positive serum is difficult to perform, as this approach easily causes optic nerve unpredictable traumas. However, Matsumoto et al. injected human NMO-IgG-positive serum directly into the space of the optic nerve sheath after desheathing, which led to infiltration of inflammatory cells into the optic nerve and regional astrocyte loss with progressive loss of RGCs and demyelination at day 14 [169].

## 11. Limitations of the Animal Models of NMO

Although each model was shown to have features of human NMO-like lesions, multiple factors may limit direct comparisons between animal and human data. First, in human NMOSD, AQP4-IgG antibodies are produced continuously, then astrocyte loss is associated with loss of myelin. Second, astrocytes and RGCs in the human CNS and eye are more complex than in rodents [176,177]. Third, the coinjection of AQP4-IgG antibodies and complement can activate the complement system in rats but not in mice and may lead to underestimation of the complement inhibitory function. Finally, EAE models in rats are Th1-cell-mediated, whereas AQP4-specific T cells in NMO reportedly show a preferential involvement of Th17/Th2 lymphocytes including IL-6- and Th17-polarizing cytokine interactions [65,108,178].

Animal models may enable us to understand the early pathogenetic mechanisms in the immune cascade of nerve inflammation and develop potential drug therapies; however, they partially recapitulate the pathological features of human NMO in animal model as the complement system and humoral/cytotoxic immunity vary between different species [170,172,179].

## 12. Conclusions

NMOSD is a complex multifactorial neuroinflammatory disease; extensive research is ongoing on the pathogenesis, genetic background, serum biomarkers, OCT segmentation. Novel drugs targeting the complement cascade system, IL-6R, and B cells are being studied. Restoring the blood–brain barrier and enhancing immune tolerance by using stem cell transplantation, dendritic cells, vaccine and regulatory T cells might provide potential therapeutic strategies in the future. Animal models may help gain a better understanding of the detailed immune mechanisms involved and could lead to the development of potential future treatments, for example, based on the inhibition of AQP4 antibody formation that could prevent the activation of inflammatory cells and offer neuroprotection.

## Figures and Tables

**Table 1:** NMOSD diagnostic criteria for adult patients.

**Diagnostic criteria for NMOSD with AQP4 IgG** At least one core clinical characteristic Positive test for AQP-IgG using an available detection method (CBA recommended) Exclusion of alternative diagnoses
**Diagnostic criteria for NMOSD without AQP4-IgG or NMOSD with unknown AQP4-IgG status** At least two core clinical characteristics occurring as a result of one or more clinical attacks and meeting all the following requirements: At least one core clinical characteristic must be optic neuritis, acute myelitis with longitudinal extensive neuritis, acute myelitis with LETM, or area postrema syndrome Dissemination in space (two or more different core clinical characteristics) Fulfillment of additional MRI criteria * Negative tests of AQP4-IgG using an available detection method, or testing unavailable Exclusion of alternative diagnoses
**Core clinical characteristics** Optic neuritis Acute myelitis Area postrema syndrome: episode of otherwise unexplained hiccups or nausea and vomiting Acute brainstem syndrome Symptomatic narcolepsy or acute diencephalic clinical syndrome with NMOSD-typical diencephalic MRI lesions Symptomatic cerebral syndrome with NMOSD-typical brain lesions
Modified IPND 2015 NMOSD Criteria [13]. * Additional MRI criteria Acute optic neuritis: requires brain MRI showing normal findings or only nonspecific white matter lesions, or optic nerve MRI with T2-hyperintense lesion or T1-weighted gadolinium-enhancing lesion extending >1/2 optic nerve length or involving optic chiasm. Acute myelitis: requires associated intramedullary MRI lesion extending ≥3 contiguous segments (LETM) OR ≥3 contiguous segments of focal spinal cord atrophy. Area postrema syndrome: requires associated dorsal medulla/area postrema lesions. Acute brainstem syndrome: requires associated periependymal brainstem lesions.

Abbreviations: NMOSD = neuromyelitis optica spectrum disorders; AQP4 = aquaporin-4; LETM = longitudinal extensive transverse myelitis; CBA = cell-based assay.

**Table 2:** Animal models of NMO-optic neuritis without experimental autoimmune encephalomyelitis.

Reference	Animal	Model System	Significance
Matsumoto et al., 2014 [169]	Adult Lewis rats	NMO patients’ sera were applied on the optic nerve after desheathing	7 days after treatment: lost expression of both AQP4 and GFAP on IHC, leading to regional astrocytic degeneration and inflammatory cell invasion, which resulted in secondary loss of RGCs and their axons
Asavapanumas et al., 2014 [42]	8- to 10-week-old, weight-matched AQP4+/+ and AQP4−/− mice in CD1 genetic background	Passive transfer of NMO-IgG and complement by continuous 3-day intracranial infusion near the optic chiasm	Loss of AQP4 and GFAP immunoreactivity, granulocyte and macrophage infiltration, deposition of activated complement, and demyelination and axonal loss
Asavapanumas et al., 2014 [161]	Adult Lewis rats	A single intracerebral needle insertion, without pre-existing inflammation or infusion of proinflammatory factors	At 5 days, there was marked loss of AQP4, GFAP, and myelin. Granulocyte and macrophage infiltration, complement deposition, BBB disruption, microglial activation, and neuron death. The penumbra was associated with a complement-independent mechanism (antibody-dependent cellular cytotoxicity).
Saadoun et al., 2010 [92]	8- to 10-week-old, wild-type and AQP4-null mice on a CD1 genetic background	Intracerebral coinjection of Ig G from NMO patients with human complement	Within 12 h of injection, striking loss of AQP4, glial cell edema, demyelination, and axonal loss, but little intraparenchymal inflammation. At 7 days, there was extensive inflammatory cell infiltration, perivascular deposition of activated complement, extensive demyelination and loss of astrocytes, and neuronal cell death.

Abbreviations: NMO = neuromyelitis optica; AQP4 = aquaporin-4; IHC = immunohistochemistry; RGCs = retinal ganglion cells; GFAP = glial fibrillary acidic protein; BBB = blood–brain barrier.

## Data Availability

Not applicable.

## References

[1] Fang C.W., Wang H.P., Chen H.M., Lin J.W., Lin W.S. (2020). Epidemiology and Comorbidities of Adult Multiple Sclerosis and Neuromyelitis Optica in Taiwan, 2001–2015. Mult. Scler. Relat. Disord..

[2] Hor J.Y., Asgari N., Nakashima I., Broadley S.A., Leite M.I., Kissani N., Jacob A., Marignier R., Weinshenker B.G., Paul F. (2020). Epidemiology of Neuromyelitis Optica Spectrum Disorder and Its Prevalence and Incidence Worldwide. Front. Neurol..

[3] Jarius S., Wildemann B. (2012). The Case of the Marquis De Causan (1804): An Early Account of Visual Loss Associated with Spinal Cord Inflammation. J. Neurol..

[4] Lennon V.A., Wingerchuk D.M., Kryzer T.J., Pittock S.J., Lucchinetti C.F., Fujihara K., Nakashima I., Weinshenker B.G. (2004). A Serum Autoantibody Marker of Neuromyelitis Optica: Distinction from Multiple Sclerosis. Lancet.

[5] Roemer S.F., Parisi J.E., Lennon V.A., Benarroch E.E., Lassmann H., Bruck W., Mandler R.N., Weinshenker B.G., Pittock S.J., Wingerchuk D.M. (2007). Pattern-Specific Loss of Aquaporin-4 Immunoreactivity Distinguishes Neuromyelitis Optica from Multiple Sclerosis. Brain.

[6] Lennon V.A., Kryzer T.J., Pittock S.J., Verkman A.S., Hinson S.R. (2005). Igg Marker of Optic-Spinal Multiple Sclerosis Binds to the Aquaporin-4 Water Channel. J. Exp. Med..

[7] Hamann S., Zeuthen T., La Cour M., Nagelhus E.A., Ottersen O.P., Agre P., Nielsen S. (1998). Aquaporins in Complex Tissues: Distribution of Aquaporins 1–5 in Human and Rat Eye. Am. J. Physiol..

[8] Nagelhus E.A., Mathiisen T.M., Ottersen O.P. (2004). Aquaporin-4 in the Central Nervous System: Cellular and Subcellular Distribution and Coexpression with Kir4.1. Neuroscience.

[9] Nagelhus E.A., Veruki M.L., Torp R., Haug F.M., Laake J.H., Nielsen S., Agre P., Ottersen O.P. (1998). Aquaporin-4 Water Channel Protein in the Rat Retina and Optic Nerve: Polarized Expression in Muller Cells and Fibrous Astrocytes. J. Neurosci..

[10] Whittam D., Wilson M., Hamid S., Keir G., Bhojak M., Jacob A. (2017). What’s New in Neuromyelitis Optica? A Short Review for the Clinical Neurologist. J. Neurol..

[11] Nielsen S., Nagelhus E.A., Amiry-Moghaddam M., Bourque C., Agre P., Ottersen O.P. (1997). Specialized Membrane Domains for Water Transport in Glial Cells: High-Resolution Immunogold Cytochemistry of Aquaporin-4 in Rat Brain. J. Neurosci..

[12] Trebst C., Jarius S., Berthele A., Paul F., Schippling S., Wildemann B., Borisow N., Kleiter I., Aktas O., Kumpfel T. (2014). Update on the Diagnosis and Treatment of Neuromyelitis Optica: Recommendations of the Neuromyelitis Optica Study Group (Nemos). J. Neurol..

[13] Wingerchuk D.M., Banwell B., Bennett J.L., Cabre P., Carroll W., Chitnis T., De Seze J., Fujihara K., Greenberg B., Jacob A. (2015). International Consensus Diagnostic Criteria for Neuromyelitis Optica Spectrum Disorders. Neurology.

[14] Hamid S.H., Elsone L., Mutch K., Solomon T., Jacob A. (2017). The Impact of 2015 Neuromyelitis Optica Spectrum Disorders Criteria on Diagnostic Rates. Mult. Scler..

[15] Li X., Zhang C., Jia D., Fan M., Li T., Tian D.C., Liu Y., Shi F.D. (2021). The Occurrence of Myelin Oligodendrocyte Glycoprotein Antibodies in Aquaporin-4-Antibody Seronegative Neuromyelitis Optica Spectrum Disorder: A Systematic Review and Meta-Analysis. Mult. Scler. Relat. Disord..

[16] Takahashi T., Fujihara K., Nakashima I., Misu T., Miyazawa I., Nakamura M., Watanabe S., Shiga Y., Kanaoka C., Fujimori J. (2007). Anti-Aquaporin-4 Antibody Is Involved in the Pathogenesis of Nmo: A Study on Antibody Titre. Brain.

[17] Schmetzer O., Lakin E., Roediger B., Duchow A., Asseyer S., Paul F., Siebert N. (2021). Anti-Aquaporin 4 Igg Is Not Associated with Any Clinical Disease Characteristics in Neuromyelitis Optica Spectrum Disorder. Front. Neurol..

[18] Rotstein D., Kim S.H., Hacohen Y., Levy M. (2021). Editorial: Epidemiology of Atypical Demyelinating Diseases. Front. Neurol..

[19] Palace J., Lin D.Y., Zeng D., Majed M., Elsone L., Hamid S., Messina S., Misu T., Sagen J., Whittam D. (2019). Outcome Prediction Models in Aqp4-Igg Positive Neuromyelitis Optica Spectrum Disorders. Brain.

[20] Kitley J., Woodhall M., Waters P., Leite M.I., Devenney E., Craig J., Palace J., Vincent A. (2012). Myelin-Oligodendrocyte Glycoprotein Antibodies in Adults with a Neuromyelitis Optica Phenotype. Neurology.

[21] Probstel A.K., Dornmair K., Bittner R., Sperl P., Jenne D., Magalhaes S., Villalobos A., Breithaupt C., Weissert R., Jacob U. (2011). Antibodies to Mog Are Transient in Childhood Acute Disseminated Encephalomyelitis. Neurology.

[22] Kaneko K., Sato D.K., Nakashima I., Nishiyama S., Tanaka S., Marignier R., Hyun J.W., Oliveira L.M., Reindl M., Seifert-Held T. (2016). Myelin Injury without Astrocytopathy in Neuroinflammatory Disorders with Mog Antibodies. J. Neurol. Neurosurg. Psychiatry.

[23] Kim H., Lee E.J., Kim S., Choi L.K., Kim K., Kim H.W., Kim K.K., Lim Y.M. (2020). Serum Biomarkers in Myelin Oligodendrocyte Glycoprotein Antibody-Associated Disease. Neurol. Neuroimmunol. Neuroinflamm..

[24] Tanaka S., Hashimoto B., Izaki S., Oji S., Fukaura H., Nomura K. (2020). Clinical and Immunological Differences between Mog Associated Disease and Anti Aqp4 Antibody-Positive Neuromyelitis Optica Spectrum Disorders: Blood-Brain Barrier Breakdown and Peripheral Plasmablasts. Mult. Scler. Relat. Disord..

[25] Fujihara K., Cook L.J. (2020). Neuromyelitis Optica Spectrum Disorders and Myelin Oligodendrocyte Glycoprotein Antibody-Associated Disease: Current Topics. Curr. Opin. Neurol..

[26] De Lott L.B., Bennett J.L., Costello F. (2022). The Changing Landscape of Optic Neuritis: A Narrative Review. J. Neurol..

[27] Khanna S., Sharma A., Huecker J., Gordon M., Naismith R.T., Van Stavern G.P. (2012). Magnetic Resonance Imaging of Optic Neuritis in Patients with Neuromyelitis Optica Versus Multiple Sclerosis. J. Neuroophthalmol..

[28] Storoni M., Davagnanam I., Radon M., Siddiqui A., Plant G.T. (2013). Distinguishing Optic Neuritis in Neuromyelitis Optica Spectrum Disease from Multiple Sclerosis: A Novel Magnetic Resonance Imaging Scoring System. J. Neuroophthalmol..

[29] Chen J.J., Flanagan E.P., Jitprapaikulsan J., Lopez-Chiriboga A.S.S., Fryer J.P., Leavitt J.A., Weinshenker B.G., Mckeon A., Tillema J.M., Lennon V.A. (2018). Myelin Oligodendrocyte Glycoprotein Antibody-Positive Optic Neuritis: Clinical Characteristics, Radiologic Clues, and Outcome. Am. J. Ophthalmol..

[30] Kitley J., Waters P., Woodhall M., Leite M.I., Murchison A., George J., Küker W., Chandratre S., Vincent A., Palace J. (2014). Neuromyelitis Optica Spectrum Disorders with Aquaporin-4 and Myelin-Oligodendrocyte Glycoprotein Antibodies: A Comparative Study. JAMA Neurol..

[31] Sato D.K., Callegaro D., Lana-Peixoto M.A., Waters P.J., De Haidar Jorge F.M., Takahashi T., Nakashima I., Apostolos-Pereira S.L., Talim N., Simm R.F. (2014). Distinction between Mog Antibody-Positive and Aqp4 Antibody-Positive Nmo Spectrum Disorders. Neurology.

[32] Jarius S., Ruprecht K., Kleiter I., Borisow N., Asgari N., Pitarokoili K., Pache F., Stich O., Beume L.A., Hummert M.W. (2016). Mog-Igg in Nmo and Related Disorders: A Multicenter Study of 50 Patients. Part 2: Epidemiology, Clinical Presentation, Radiological and Laboratory Features, Treatment Responses, and Long-Term Outcome. J. Neuroinflamm..

[33] Jiao Y., Fryer J.P., Lennon V.A., Jenkins S.M., Quek A.M., Smith C.Y., Mckeon A., Costanzi C., Iorio R., Weinshenker B.G. (2013). Updated Estimate of Aqp4-Igg Serostatus and Disability Outcome in Neuromyelitis Optica. Neurology.

[34] Selmaj K., Selmaj I. (2019). Novel Emerging Treatments for Nmosd. Neurol. Neurochir. Pol..

[35] Traboulsee A., Greenberg B.M., Bennett J.L., Szczechowski L., Fox E., Shkrobot S., Yamamura T., Terada Y., Kawata Y., Wright P. (2020). Safety and Efficacy of Satralizumab Monotherapy in Neuromyelitis Optica Spectrum Disorder: A Randomised, Double-Blind, Multicentre, Placebo-Controlled Phase 3 Trial. Lancet Neurol..

[36] Cree B.a.C., Bennett J.L., Kim H.J., Weinshenker B.G., Pittock S.J., Wingerchuk D.M., Fujihara K., Paul F., Cutter G.R., Marignier R. (2019). Inebilizumab for the Treatment of Neuromyelitis Optica Spectrum Disorder (N-Momentum): A Double-Blind, Randomised Placebo-Controlled Phase 2/3 Trial. Lancet.

[37] Wingerchuk D.M., Zhang I., Kielhorn A., Royston M., Levy M., Fujihara K., Nakashima I., Tanvir I., Paul F., Pittock S.J. (2022). Network Meta-Analysis of Food and Drug Administration-Approved Treatment Options for Adults with Aquaporin-4 Immunoglobulin G-Positive Neuromyelitis Optica Spectrum Disorder. Neurol. Ther..

[38] Pittock S.J., Lennon V.A., Mckeon A., Mandrekar J., Weinshenker B.G., Lucchinetti C.F., O’toole O., Wingerchuk D.M. (2013). Eculizumab in Aqp4-Igg-Positive Relapsing Neuromyelitis Optica Spectrum Disorders: An Open-Label Pilot Study. Lancet Neurol..

[39] Katz Sand I., Fabian M.T., Telford R., Kraus T.A., Chehade M., Masilamani M., Moran T., Farrell C., Ebel S., Cook L.J. (2018). Open-Label, Add-on Trial of Cetirizine for Neuromyelitis Optica. Neurol. Neuroimmunol. Neuroinflamm..

[40] Herges K., De Jong B.A., Kolkowitz I., Dunn C., Mandelbaum G., Ko R.M., Maini A., Han M.H., Killestein J., Polman C. (2012). Protective Effect of an Elastase Inhibitor in a Neuromyelitis Optica-Like Disease Driven by a Peptide of Myelin Oligodendroglial Glycoprotein. Mult. Scler..

[41] Ransom B., Behar T., Nedergaard M. (2003). New Roles for Astrocytes (Stars at Last). Trends Neurosci..

[42] Asavapanumas N., Ratelade J., Papadopoulos M.C., Bennett J.L., Levin M.H., Verkman A.S. (2014). Experimental Mouse Model of Optic Neuritis with Inflammatory Demyelination Produced by Passive Transfer of Neuromyelitis Optica-Immunoglobulin G. J. Neuroinflamm..

[43] Bennett J.L., De Seze J., Lana-Peixoto M., Palace J., Waldman A., Schippling S., Tenembaum S., Banwell B., Greenberg B., Levy M. (2015). Neuromyelitis Optica and Multiple Sclerosis: Seeing Differences through Optical Coherence Tomography. Mult. Scler..

[44] Lucchinetti C.F., Mandler R.N., Mcgavern D., Bruck W., Gleich G., Ransohoff R.M., Trebst C., Weinshenker B., Wingerchuk D., Parisi J.E. (2002). A Role for Humoral Mechanisms in the Pathogenesis of Devic’s Neuromyelitis Optica. Brain.

[45] Lucchinetti C.F., Guo Y., Popescu B.F.G., Fujihara K., Itoyama Y., Misu T. (2014). The Pathology of an Autoimmune Astrocytopathy: Lessons Learned from Neuromyelitis Optica. Brain Pathol..

[46] Hinson S.R., Romero M.F., Popescu B.F., Lucchinetti C.F., Fryer J.P., Wolburg H., Fallier-Becker P., Noell S., Lennon V.A. (2012). Molecular Outcomes of Neuromyelitis Optica (Nmo)-Igg Binding to Aquaporin-4 in Astrocytes. Proc. Natl. Acad. Sci. USA.

[47] Crane J.M., Tajima M., Verkman A.S. (2010). Live-Cell Imaging of Aquaporin-4 Diffusion and Interactions in Orthogonal Arrays of Particles. Neuroscience.

[48] Abe Y., Yasui M. (2022). Aquaporin-4 in Neuromyelitis Optica Spectrum Disorders: A Target of Autoimmunity in the Central Nervous System. Biomolecules.

[49] Mckeon A., Lennon V.A., Lotze T., Tenenbaum S., Ness J.M., Rensel M., Kuntz N.L., Fryer J.P., Homburger H., Hunter J. (2008). Cns Aquaporin-4 Autoimmunity in Children. Neurology.

[50] Matiello M., Schaefer-Klein J., Sun D., Weinshenker B.G. (2013). Aquaporin 4 Expression and Tissue Susceptibility to Neuromyelitis Optica. JAMA Neurol..

[51] Crane J.M., Van Hoek A.N., Skach W.R., Verkman A.S. (2008). Aquaporin-4 Dynamics in Orthogonal Arrays in Live Cells Visualized by Quantum Dot Single Particle Tracking. Mol. Biol. Cell.

[52] Neely J.D., Christensen B.M., Nielsen S., Agre P. (1999). Heterotetrameric Composition of Aquaporin-4 Water Channels. Biochemistry.

[53] Phuan P.W., Ratelade J., Rossi A., Tradtrantip L., Verkman A.S. (2012). Complement-Dependent Cytotoxicity in Neuromyelitis Optica Requires Aquaporin-4 Protein Assembly in Orthogonal Arrays. J. Biol. Chem..

[54] Li J., Bazzi S.A., Schmitz F., Tanno H., Mcdaniel J.R., Lee C.-H., Joshi C., Kim J.E., Monson N., Greenberg B.M. (2021). Molecular Level Characterization of Circulating Aquaporin-4 Antibodies in Neuromyelitis Optica Spectrum Disorder. Neurol. Neuroimmunol. Neuroinflamm..

[55] Zeka B., Lassmann H., Bradl M. (2017). Muller Cells and Retinal Axons Can Be Primary Targets in Experimental Neuromyelitis Optica Spectrum Disorder. Clin. Exp. Neuroimmunol..

[56] Wingerchuk D.M. (2013). Neuromyelitis Optica: Potential Roles for Intravenous Immunoglobulin. J. Clin. Immunol..

[57] You Y., Zhu L., Zhang T., Shen T., Fontes A., Yiannikas C., Parratt J., Barton J., Schulz A., Gupta V. (2019). Evidence of Müller Glial Dysfunction in Patients with Aquaporin-4 Immunoglobulin G-Positive Neuromyelitis Optica Spectrum Disorder. Ophthalmology.

[58] Ratelade J., Asavapanumas N., Ritchie A.M., Wemlinger S., Bennett J.L., Verkman A.S. (2013). Involvement of Antibody-Dependent Cell-Mediated Cytotoxicity in Inflammatory Demyelination in a Mouse Model of Neuromyelitis Optica. Acta Neuropathol..

[59] Duan T., Smith A.J., Verkman A.S. (2019). Complement-Independent Bystander Injury in Aqp4-Igg Seropositive Neuromyelitis Optica Produced by Antibody-Dependent Cellular Cytotoxicity. Acta Neuropathol. Commun..

[60] Asavapanumas N., Ratelade J., Verkman A.S. (2014). Unique Neuromyelitis Optica Pathology Produced in Naive Rats by Intracerebral Administration of Nmo-Igg. Acta Neuropathol..

[61] Seidel U.J., Schlegel P., Lang P. (2013). Natural Killer Cell Mediated Antibody-Dependent Cellular Cytotoxicity in Tumor Immunotherapy with Therapeutic Antibodies. Front. Immunol..

[62] Imai K., Takaoka A. (2006). Comparing Antibody and Small-Molecule Therapies for Cancer. Nat. Rev. Cancer.

[63] Huppert J., Closhen D., Croxford A., White R., Kulig P., Pietrowski E., Bechmann I., Becher B., Luhmann H.J., Waisman A. (2010). Cellular Mechanisms of Il-17-Induced Blood-Brain Barrier Disruption. FASEB J..

[64] Carlson T., Kroenke M., Rao P., Lane T.E., Segal B. (2008). The Th17–Elr+ Cxc Chemokine Pathway Is Essential for the Development of Central Nervous System Autoimmune Disease. J. Exp. Med..

[65] Varrin-Doyer M., Spencer C.M., Schulze-Topphoff U., Nelson P.A., Stroud R.M., Cree B.A.C., Zamvil S.S. (2012). Aquaporin 4-Specific T Cells in Neuromyelitis Optica Exhibit a Th17 Bias and Recognize Clostridium Abc Transporter. Ann. Neurol..

[66] Uzawa A., Mori M., Arai K., Sato Y., Hayakawa S., Masuda S., Taniguchi J., Kuwabara S. (2010). Cytokine and Chemokine Profiles in Neuromyelitis Optica: Significance of Interleukin-6. Mult. Scler. J..

[67] Passos G.R.D., Sato D.K., Becker J., Fujihara K. (2016). Th17 Cells Pathways in Multiple Sclerosis and Neuromyelitis Optica Spectrum Disorders: Pathophysiological and Therapeutic Implications. Mediat. Inflamm..

[68] Chen D., Gallagher S., Monson N.L., Herbst R., Wang Y. (2016). Inebilizumab, a B Cell-Depleting Anti-Cd19 Antibody for the Treatment of Autoimmune Neurological Diseases: Insights from Preclinical Studies. J. Clin. Med..

[69] Challa D.K., Bussmeyer U., Khan T., Montoyo H.P., Bansal P., Ober R.J., Ward E.S. (2013). Autoantibody Depletion Ameliorates Disease in Murine Experimental Autoimmune Encephalomyelitis. mAbs.

[70] Hinson S.R., Pittock S.J., Lucchinetti C.F., Roemer S.F., Fryer J.P., Kryzer T.J., Lennon V.A. (2007). Pathogenic Potential of Igg Binding to Water Channel Extracellular Domain in Neuromyelitis Optica. Neurology.

[71] Yao X., Verkman A.S. (2017). Complement Regulator Cd59 Prevents Peripheral Organ Injury in Rats Made Seropositive for Neuromyelitis Optica Immunoglobulin G. Acta Neuropathol. Commun..

[72] Soltys J., Liu Y., Ritchie A., Wemlinger S., Schaller K., Schumann H., Owens G.P., Bennett J.L. (2019). Membrane Assembly of Aquaporin-4 Autoantibodies Regulates Classical Complement Activation in Neuromyelitis Optica. J. Clin. Investig..

[73] Phuan P.W., Zhang H., Asavapanumas N., Leviten M., Rosenthal A., Tradtrantip L., Verkman A.S. (2013). C1q-Targeted Monoclonal Antibody Prevents Complement-Dependent Cytotoxicity and Neuropathology in In Vitro and Mouse Models of Neuromyelitis Optica. Acta Neuropathol..

[74] Brachet G., Bourquard T., Gallay N., Reiter E., Gouilleux-Gruart V., Poupon A., Watier H. (2016). Eculizumab Epitope on Complement C5: Progress towards a Better Understanding of the Mechanism of Action. Mol. Immunol..

[75] Hillmen P., Young N.S., Schubert J., Brodsky R.A., Socie G., Muus P., Roth A., Szer J., Elebute M.O., Nakamura R. (2006). The Complement Inhibitor Eculizumab in Paroxysmal Nocturnal Hemoglobinuria. N. Engl. J. Med..

[76] Merle N.S., Noe R., Halbwachs-Mecarelli L., Fremeaux-Bacchi V., Roumenina L.T. (2015). Complement System Part II: Role in Immunity. Front. Immunol..

[77] Pache F., Ringelstein M., Aktas O., Kleiter I., Jarius S., Siebert N., Bellmann-Strobl J., Paul F., Ruprecht K. (2021). C3 and C4 Complement Levels in Aqp4-Igg-Positive Nmosd and in Mogad. J. Neuroimmunol..

[78] Takai Y., Misu T., Kaneko K., Chihara N., Narikawa K., Tsuchida S., Nishida H., Komori T., Seki M., Komatsu T. (2020). Myelin Oligodendrocyte Glycoprotein Antibody-Associated Disease: An Immunopathological Study. Brain.

[79] Kaneko K., Sato D.K., Nakashima I., Ogawa R., Akaishi T., Takai Y., Nishiyama S., Takahashi T., Misu T., Kuroda H. (2018). Csf Cytokine Profile in Mog-Igg+ Neurological Disease Is Similar to Aqp4-Igg+ Nmosd but Distinct from Ms: A Cross-Sectional Study and Potential Therapeutic Implications. J. Neurol. Neurosurg. Psychiatry.

[80] Saadoun S., Waters P., Owens G.P., Bennett J.L., Vincent A., Papadopoulos M.C. (2014). Neuromyelitis Optica Mog-Igg Causes Reversible Lesions in Mouse Brain. Acta Neuropathol. Commun..

[81] Peschl P., Schanda K., Zeka B., Given K., Bohm D., Ruprecht K., Saiz A., Lutterotti A., Rostasy K., Hoftberger R. (2017). Human Antibodies against the Myelin Oligodendrocyte Glycoprotein Can Cause Complement-Dependent Demyelination. J. Neuroinflamm..

[82] Keller C.W., Lopez J.A., Wendel E.M., Ramanathan S., Gross C.C., Klotz L., Reindl M., Dale R.C., Wiendl H., Rostásy K. (2021). Complement Activation Is a Prominent Feature of Mogad. Ann. Neurol..

[83] Carnero Contentti E., Correale J. (2021). Neuromyelitis Optica Spectrum Disorders: From Pathophysiology to Therapeutic Strategies. J. Neuroinflamm..

[84] Papadopoulos M.C., Bennett J.L., Verkman A.S. (2014). Treatment of Neuromyelitis Optica: State-of-the-Art and Emerging Therapies. Nat. Rev. Neurol..

[85] Zhang P., Liu B. (2020). Effect of Autologous Hematopoietic Stem Cell Transplantation on Multiple Sclerosis and Neuromyelitis Optica Spectrum Disorder: A Prisma-Compliant Meta-Analysis. Bone Marrow Transplant..

[86] Zubizarreta I., Flórez-Grau G., Vila G., Cabezón R., España C., Andorra M., Saiz A., Llufriu S., Sepulveda M., Sola-Valls N. (2019). Immune Tolerance in Multiple Sclerosis and Neuromyelitis Optica with Peptide-Loaded Tolerogenic Dendritic Cells in a Phase 1b Trial. Proc. Natl. Acad. Sci. USA.

[87] Garren H., Robinson W.H., Krasulová E., Havrdová E., Nadj C., Selmaj K., Losy J., Nadj I., Radue E.W., Kidd B.A. (2008). Phase 2 Trial of a DNA Vaccine Encoding Myelin Basic Protein for Multiple Sclerosis. Ann. Neurol..

[88] Bar-Or A., Steinman L., Behne J.M., Benitez-Ribas D., Chin P.S., Clare-Salzler M., Healey D., Kim J.I., Kranz D.M., Lutterotti A. (2016). Restoring Immune Tolerance in Neuromyelitis Optica: Part II. Neurol. Neuroimmunol. Neuroinflamm..

[89] Steinman L., Bar-Or A., Behne J.M., Benitez-Ribas D., Chin P.S., Clare-Salzler M., Healey D., Kim J.I., Kranz D.M., Lutterotti A. (2016). Restoring Immune Tolerance in Neuromyelitis Optica: Part I. Neurol. Neuroimmunol. Neuroinflamm..

[90] Shimizu F., Nishihara H., Kanda T. (2018). Blood-Brain Barrier Dysfunction in Immuno-Mediated Neurological Diseases. Immunol. Med..

[91] Asavapanumas N., Tradtrantip L., Verkman A.S. (2021). Targeting the Complement System in Neuromyelitis Optica Spectrum Disorder. Expert Opin. Biol. Ther..

[92] Saadoun S., Waters P., Bell B.A., Vincent A., Verkman A.S., Papadopoulos M.C. (2010). Intra-Cerebral Injection of Neuromyelitis Optica Immunoglobulin G and Human Complement Produces Neuromyelitis Optica Lesions in Mice. Brain.

[93] Ghafouri-Fard S., Azimi T., Taheri M. (2021). A Comprehensive Review on the Role of Genetic Factors in Neuromyelitis Optica Spectrum Disorder. Front. Immunol..

[94] Matiello M., Kim H.J., Kim W., Brum D.G., Barreira A.A., Kingsbury D.J., Plant G.T., Adoni T., Weinshenker B.G. (2010). Familial Neuromyelitis Optica. Neurology.

[95] Zhong X., Chen C., Sun X., Wang J., Li R., Chang Y., Fan P., Wang Y., Wu Y., Peng L. (2021). Whole-Exome Sequencing Reveals the Major Genetic Factors Contributing to Neuromyelitis Optica Spectrum Disorder in Chinese Patients with Aquaporin 4-Igg Seropositivity. Eur. J. Neurol..

[96] Zéphir H., Fajardy I., Outteryck O., Blanc F., Roger N., Fleury M., Rudolf G., Marignier R., Vukusic S., Confavreux C. (2009). Is Neuromyelitis Optica Associated with Human Leukocyte Antigen?. Mult. Scler..

[97] Matsushita T., Masaki K., Isobe N., Sato S., Yamamoto K., Nakamura Y., Watanabe M., Suenaga T., Kira J.I. (2020). Genetic Factors for Susceptibility to and Manifestations of Neuromyelitis Optica. Ann. Clin. Transl. Neurol..

[98] Estrada K., Whelan C.W., Zhao F., Bronson P., Handsaker R.E., Sun C., Carulli J.P., Harris T., Ransohoff R.M., Mccarroll S.A. (2018). A Whole-Genome Sequence Study Identifies Genetic Risk Factors for Neuromyelitis Optica. Nat. Commun..

[99] Ogawa K., Okuno T., Hosomichi K., Hosokawa A., Hirata J., Suzuki K., Sakaue S., Kinoshita M., Asano Y., Miyamoto K. (2019). Next-Generation Sequencing Identifies Contribution of Both Class I and Ii Hla Genes on Susceptibility of Multiple Sclerosis in Japanese. J. Neuroinflamm..

[100] Beppu S., Kinoshita M., Wilamowski J., Suenaga T., Yasumizu Y., Ogawa K., Ishikura T., Tada S., Koda T., Murata H. (2022). High Cell Surface Expression and Peptide Binding Affinity of Hla-Dqa1*05:03, a Susceptible Allele of Neuromyelitis Optica Spectrum Disorders (Nmosd). Sci. Rep..

[101] Watanabe M., Nakamura Y., Sato S., Niino M., Fukaura H., Tanaka M., Ochi H., Kanda T., Takeshita Y., Yokota T. (2021). Hla Genotype-Clinical Phenotype Correlations in Multiple Sclerosis and Neuromyelitis Optica Spectrum Disorders Based on Japan Ms/Nmosd Biobank Data. Sci. Rep..

[102] Yoshimura S., Isobe N., Matsushita T., Yonekawa T., Masaki K., Sato S., Kawano Y., Kira J. (2013). Distinct Genetic and Infectious Profiles in Japanese Neuromyelitis Optica Patients According to Anti-Aquaporin 4 Antibody Status. J. Neurol. Neurosurg. Psychiatry.

[103] Li T., Li H., Li Y., Dong S.A., Yi M., Zhang Q.X., Feng B., Yang L., Shi F.D., Yang C.S. (2021). Multi-Level Analyses of Genome-Wide Association Study to Reveal Significant Risk Genes and Pathways in Neuromyelitis Optica Spectrum Disorder. Front. Genet..

[104] Uzawa A., Mori M., Kuwabara S. (2014). Cytokines and Chemokines in Neuromyelitis Optica: Pathogenetic and Therapeutic Implications. Brain Pathol..

[105] Rocca M.A., Cacciaguerra L., Filippi M. (2020). Moving Beyond Anti-Aquaporin-4 Antibodies: Emerging Biomarkers in the Spectrum of Neuromyelitis Optica. Expert Rev. Neurother..

[106] Park H., Li Z., Yang X.O., Chang S.H., Nurieva R., Wang Y.-H., Wang Y., Hood L., Zhu Z., Tian Q. (2005). A Distinct Lineage of Cd4 T Cells Regulates Tissue Inflammation by Producing Interleukin 17. Nat. Immunol..

[107] Hou M.M., Li Y.F., He L.L., Li X.Q., Zhang Y., Zhang S.X., Li X.Y. (2019). Proportions of Th17 Cells and Th17-Related Cytokines in Neuromyelitis Optica Spectrum Disorders Patients: A Meta-Analysis. Int. Immunopharmacol..

[108] Maciak K., Pietrasik S., Dziedzic A., Redlicka J., Saluk-Bijak J., Bijak M., Włodarczyk T., Miller E. (2021). Th17-Related Cytokines as Potential Discriminatory Markers between Neuromyelitis Optica (Devic’s Disease) and Multiple Sclerosis—A Review. Int. J. Mol. Sci..

[109] Fujihara K., Bennett J.L., De Seze J., Haramura M., Kleiter I., Weinshenker B.G., Kang D., Mughal T., Yamamura T. (2020). Interleukin-6 in Neuromyelitis Optica Spectrum Disorder Pathophysiology. Neurol. Neuroimmunol. Neuroinflamm..

[110] Takeshita Y., Fujikawa S., Serizawa K., Fujisawa M., Matsuo K., Nemoto J., Shimizu F., Sano Y., Tomizawa-Shinohara H., Miyake S. (2021). New Bbb Model Reveals That Il-6 Blockade Suppressed the Bbb Disorder, Preventing Onset of Nmosd. Neurol. Neuroimmunol. Neuroinflamm..

[111] Langrish C.L., Chen Y., Blumenschein W.M., Mattson J., Basham B., Sedgwick J.D., Mcclanahan T., Kastelein R.A., Cua D.J. (2005). Il-23 Drives a Pathogenic T Cell Population That Induces Autoimmune Inflammation. J. Exp. Med..

[112] Korn T., Bettelli E., Oukka M., Kuchroo V.K. (2009). Il-17 and Th17 Cells. Annu. Rev. Immunol..

[113] El-Behi M., Ciric B., Dai H., Yan Y., Cullimore M., Safavi F., Zhang G.-X., Dittel B.N., Rostami A. (2011). The Encephalitogenicity of Th17 Cells Is Dependent on Il-1- and Il-23-Induced Production of the Cytokine Gm-Csf. Nat. Immunol..

[114] Monteiro C., Fernandes G., Kasahara T.M., Barros P.O., Dias A.S.O., Araujo A., Ornelas A.M.M., Aguiar R.S., Alvarenga R., Bento C.a.M. (2019). The Expansion of Circulating Il-6 and Il-17-Secreting Follicular Helper T Cells Is Associated with Neurological Disabilities in Neuromyelitis Optica Spectrum Disorders. J. Neuroimmunol..

[115] Uchida T., Mori M., Uzawa A., Masuda H., Muto M., Ohtani R., Kuwabara S. (2017). Increased Cerebrospinal Fluid Metalloproteinase-2 and Interleukin-6 Are Associated with Albumin Quotient in Neuromyelitis Optica: Their Possible Role on Blood-Brain Barrier Disruption. Mult. Scler..

[116] Kimura A., Naka T., Kishimoto T. (2007). Il-6-Dependent and -Independent Pathways in the Development of Interleukin 17-Producing T Helper Cells. Proc. Natl. Acad. Sci. USA.

[117] Takeshita Y., Obermeier B., Cotleur A.C., Spampinato S.F., Shimizu F., Yamamoto E., Sano Y., Kryzer T.J., Lennon V.A., Kanda T. (2017). Effects of Neuromyelitis Optica-Igg at the Blood-Brain Barrier in Vitro. Neurol. Neuroimmunol. Neuroinflamm..

[118] Uzawa A., Mori M., Kuwabara S. (2013). Role of Interleukin-6 in the Pathogenesis of Neuromyelitis Optica. Clin. Exp. Neuroimmunol..

[119] Kaplin A.I., Deshpande D.M., Scott E., Krishnan C., Carmen J.S., Shats I., Martinez T., Drummond J., Dike S., Pletnikov M. (2005). Il-6 Induces Regionally Selective Spinal Cord Injury in Patients with the Neuroinflammatory Disorder Transverse Myelitis. J. Clin. Investig..

[120] Moinfar Z., Zamvil S.S. (2020). Microglia Complement Astrocytes in Neuromyelitis Optica. J. Clin. Investig..

[121] Li W., Liu J., Tan W., Zhou Y. (2021). The Role and Mechanisms of Microglia in Neuromyelitis Optica Spectrum Disorders. Int. J. Med. Sci..

[122] Takano R., Misu T., Takahashi T., Sato S., Fujihara K., Itoyama Y. (2010). Astrocytic Damage Is Far More Severe Than Demyelination in Nmo: A Clinical Csf Biomarker Study. Neurology.

[123] Wang J., Cui C., Lu Y., Chang Y., Wang Y., Li R., Shan Y., Sun X., Long Y., Wang H. (2021). Therapeutic Response and Possible Biomarkers in Acute Attacks of Neuromyelitis Optica Spectrum Disorders: A Prospective Observational Study. Front. Immunol..

[124] Watanabe M., Nakamura Y., Michalak Z., Isobe N., Barro C., Leppert D., Matsushita T., Hayashi F., Yamasaki R., Kuhle J. (2019). Serum Gfap and Neurofilament Light as Biomarkers of Disease Activity and Disability in Nmosd. Neurology.

[125] Aktas O., Smith M.A., Rees W.A., Bennett J.L., She D., Katz E., Cree B.a.C., N-MOmentum Scientific Group and the N-MOmentum Study Investigators (2021). Serum Glial Fibrillary Acidic Protein: A Neuromyelitis Optica Spectrum Disorder Biomarker. Ann. Neurol..

[126] Schindler P., Grittner U., Oechtering J., Leppert D., Siebert N., Duchow A.S., Oertel F.C., Asseyer S., Kuchling J., Zimmermann H.G. (2021). Serum Gfap and Nfl as Disease Severity and Prognostic Biomarkers in Patients with Aquaporin-4 Antibody-Positive Neuromyelitis Optica Spectrum Disorder. J. Neuroinflamm..

[127] Fu C.C., Gao C., Zhang H.H., Mao Y.Q., Lu J.Q., Petritis B., Huang A.S., Yang X.G., Long Y.M., Huang R.P. (2022). Serum Molecular Biomarkers in Neuromyelitis Optica and Multiple Sclerosis. Mult. Scler. Relat. Disord..

[128] Sonar S.A., Shaikh S., Joshi N., Atre A.N., Lal G. (2017). Ifn-Gamma Promotes Transendothelial Migration of Cd4^+^ T Cells across the Blood-Brain Barrier. Immunol. Cell Biol..

[129] Bonney S., Seitz S., Ryan C.A., Jones K.L., Clarke P., Tyler K.L., Siegenthaler J.A. (2019). Gamma Interferon Alters Junctional Integrity Via Rho Kinase, Resulting in Blood-Brain Barrier Leakage in Experimental Viral Encephalitis. mBio.

[130] Tong Y., Yang T., Wang J., Zhao T., Wang L., Kang Y., Cheng C., Fan Y. (2018). Elevated Plasma Chemokines for Eosinophils in Neuromyelitis Optica Spectrum Disorders during Remission. Front. Neurol..

[131] Specovius S., Zimmermann H.G., Oertel F.C., Chien C., Bereuter C., Cook L.J., Lana Peixoto M.A., Fontenelle M.A., Kim H.J., Hyun J.W. (2020). Cohort Profile: A Collaborative Multicentre Study of Retinal Optical Coherence Tomography in 539 Patients with Neuromyelitis Optica Spectrum Disorders (Croctino). BMJ Open.

[132] Oertel F.C., Specovius S., Zimmermann H.G., Chien C., Motamedi S., Bereuter C., Cook L., Lana Peixoto M.A., Fontanelle M.A., Kim H.J. (2021). Retinal Optical Coherence Tomography in Neuromyelitis Optica. Neurol. Neuroimmunol. Neuroinflamm..

[133] Graves J.S., Oertel F.C., Van Der Walt A., Collorone S., Sotirchos E.S., Pihl-Jensen G., Albrecht P., Yeh E.A., Saidha S., Frederiksen J. (2022). Leveraging Visual Outcome Measures to Advance Therapy Development in Neuroimmunologic Disorders. Neurol. Neuroimmunol. Neuroinflamm..

[134] Schneider E., Zimmermann H., Oberwahrenbrock T., Kaufhold F., Kadas E.M., Petzold A., Bilger F., Borisow N., Jarius S., Wildemann B. (2013). Optical Coherence Tomography Reveals Distinct Patterns of Retinal Damage in Neuromyelitis Optica and Multiple Sclerosis. PLoS ONE.

[135] Wingerchuk D.M., Pittock S.J., Lucchinetti C.F., Lennon V.A., Weinshenker B.G. (2007). A Secondary Progressive Clinical Course Is Uncommon in Neuromyelitis Optica. Neurology.

[136] Sotirchos E.S., Saidha S., Byraiah G., Mealy M.A., Ibrahim M.A., Sepah Y.J., Newsome S.D., Ratchford J.N., Frohman E.M., Balcer L.J. (2013). In Vivo Identification of Morphologic Retinal Abnormalities in Neuromyelitis Optica. Neurology.

[137] Oertel F.C., Kuchling J., Zimmermann H., Chien C., Schmidt F., Knier B., Bellmann-Strobl J., Korn T., Scheel M., Klistorner A. (2017). Microstructural Visual System Changes in Aqp4-Antibody–Seropositive Nmosd. Neurol. Neuroimmunol. Neuroinflamm..

[138] Van Horssen J., Witte M.E., Ciccarelli O. (2012). The Role of Mitochondria in Axonal Degeneration and Tissue Repair in Ms. Mult. Scler..

[139] Evangelou N., Konz D., Esiri M.M., Smith S., Palace J., Matthews P.M. (2001). Size-Selective Neuronal Changes in the Anterior Optic Pathways Suggest a Differential Susceptibility to Injury in Multiple Sclerosis. Brain.

[140] Al-Nosairy K.O., Horbrügger M., Schippling S., Wagner M., Haghikia A., Pawlitzki M., Hoffmann M.B. (2021). Structure–Function Relationship of Retinal Ganglion Cells in Multiple Sclerosis. Int. J. Mol. Sci..

[141] La Morgia C., Di Vito L., Carelli V., Carbonelli M. (2017). Patterns of Retinal Ganglion Cell Damage in Neurodegenerative Disorders: Parvocellular vs Magnocellular Degeneration in Optical Coherence Tomography Studies. Front. Neurol..

[142] Zeng P., Du C., Zhang R., Jia D., Jiang F., Fan M., Zhang C. (2021). Optical Coherence Tomography Reveals Longitudinal Changes in Retinal Damage under Different Treatments for Neuromyelitis Optica Spectrum Disorder. Front. Neurol..

[143] Jeong I.H., Kim H.J., Kim N.H., Jeong K.S., Park C.Y. (2016). Subclinical Primary Retinal Pathology in Neuromyelitis Optica Spectrum Disorder. J. Neurol..

[144] Roca-Fernandez A., Oertel F.C., Yeo T., Motamedi S., Probert F., Craner M.J., Sastre-Garriga J., Zimmermann H.G., Asseyer S., Kuchling J. (2021). Foveal Changes in Aquaporin-4 Antibody Seropositive Neuromyelitis Optica Spectrum Disorder Are Independent of Optic Neuritis and Not Overtly Progressive. Eur. J. Neurol..

[145] Felix C.M., Levin M.H., Verkman A.S. (2016). Complement-Independent Retinal Pathology Produced by Intravitreal Injection of Neuromyelitis Optica Immunoglobulin G. J. Neuroinflamm..

[146] Chen X., Kuehlewein L., Pineles S.L., Tandon A.K., Bose S.X., Klufas M.A., Sadda S.R., Sarraf D. (2015). En Face Optical Coherence Tomography of Macular Microcysts Due to Optic Neuropathy from Neuromyelitis Optica. Retin Cases Brief Rep..

[147] Wolff B., Basdekidou C., Vasseur V., Mauget-Faysse M., Sahel J.A., Vignal C. (2013). Retinal Inner Nuclear Layer Microcystic Changes in Optic Nerve Atrophy: A Novel Spectral-Domain Oct Finding. Retina.

[148] Zhang S., Wang H., Lu Q., Qing G., Wang N., Wang Y., Li S., Yang D., Yan F. (2009). Detection of Early Neuron Degeneration and Accompanying Glial Responses in the Visual Pathway in a Rat Model of Acute Intraocular Hypertension. Brain Res..

[149] Mirza R.G., Johnson M.W., Jampol L.M. (2007). Optical Coherence Tomography Use in Evaluation of the Vitreoretinal Interface: A Review. Surv. Ophthalmol..

[150] Gelfand J.M., Nolan R., Schwartz D.M., Graves J., Green A.J. (2012). Microcystic Macular Oedema in Multiple Sclerosis Is Associated with Disease Severity. Brain.

[151] Kappos L., Radue E.W., O’connor P., Polman C., Hohlfeld R., Calabresi P., Selmaj K., Agoropoulou C., Leyk M., Zhang-Auberson L. (2010). A Placebo-Controlled Trial of Oral Fingolimod in Relapsing Multiple Sclerosis. N. Engl. J. Med..

[152] Barboni P., Carelli V., Savini G., Carbonelli M., La Morgia C., Sadun A.A. (2013). Microcystic Macular Degeneration from Optic Neuropathy: Not Inflammatory, Not Trans-Synaptic Degeneration. Brain.

[153] Strong S.A., Hirji N., Quartilho A., Kalitzeos A., Michaelides M. (2019). Retrospective Cohort Study Exploring Whether an Association Exists between Spatial Distribution of Cystoid Spaces in Cystoid Macular Oedema Secondary to Retinitis Pigmentosa and Response to Treatment with Carbonic Anhydrase Inhibitors. Br. J. Ophthalmol..

[154] Hajali M., Fishman G.A., Anderson R.J. (2008). The Prevalence of Cystoid Macular Oedema in Retinitis Pigmentosa Patients Determined by Optical Coherence Tomography. Br. J. Ophthalmol..

[155] Abegg M., Zinkernagel M., Wolf S. (2012). Microcystic Macular Degeneration from Optic Neuropathy. Brain.

[156] Balk L.J., Killestein J., Polman C.H., Uitdehaag B.M., Petzold A. (2012). Microcystic Macular Oedema Confirmed, but Not Specific for Multiple Sclerosis. Brain.

[157] Agte S., Junek S., Matthias S., Ulbricht E., Erdmann I., Wurm A., Schild D., Käs J.A., Reichenbach A. (2011). Müller Glial Cell-Provided Cellular Light Guidance through the Vital Guinea-Pig Retina. Biophys. J..

[158] Aly L., Strauß E.M., Feucht N., Weiß I., Berthele A., Mitsdoerffer M., Haass C., Hemmer B., Maier M., Korn T. (2021). Optical Coherence Tomography Angiography Indicates Subclinical Retinal Disease in Neuromyelitis Optica Spectrum Disorders. Mult. Scler..

[159] Lin T.-Y., Chien C., Lu A., Paul F., Zimmermann H.G. (2021). Retinal Optical Coherence Tomography and Magnetic Resonance Imaging in Neuromyelitis Optica Spectrum Disorders and Mog-Antibody Associated Disorders: An Updated Review. Expert Rev. Neurother..

[160] Kwapong W.R., Peng C., He Z., Zhuang X., Shen M., Lu F. (2018). Altered Macular Microvasculature in Neuromyelitis Optica Spectrum Disorders. Am. J. Ophthalmol..

[161] Asavapanumas N., Verkman A.S. (2014). Neuromyelitis Optica Pathology in Rats following Intraperitoneal Injection of Nmo-Igg and Intracerebral Needle Injury. Acta Neuropathol. Commun..

[162] Kinoshita M., Nakatsuji Y., Kimura T., Moriya M., Takata K., Okuno T., Kumanogoh A., Kajiyama K., Yoshikawa H., Sakoda S. (2009). Neuromyelitis Optica: Passive Transfer to Rats by Human Immunoglobulin. Biochem. Biophys. Res. Commun..

[163] Kurosawa K., Misu T., Takai Y., Sato D.K., Takahashi T., Abe Y., Iwanari H., Ogawa R., Nakashima I., Fujihara K. (2015). Severely Exacerbated Neuromyelitis Optica Rat Model with Extensive Astrocytopathy by High Affinity Anti-Aquaporin-4 Monoclonal Antibody. Acta Neuropathol. Commun..

[164] Bennett J.L., Lam C., Kalluri S.R., Saikali P., Bautista K., Dupree C., Glogowska M., Case D., Antel J.P., Owens G.P. (2009). Intrathecal Pathogenic Anti-Aquaporin-4 Antibodies in Early Neuromyelitis Optica. Ann. Neurol..

[165] Bradl M., Lassmann H. (2014). Experimental Models of Neuromyelitis Optica. Brain Pathol..

[166] Bradl M., Misu T., Takahashi T., Watanabe M., Mader S., Reindl M., Adzemovic M., Bauer J., Berger T., Fujihara K. (2009). Neuromyelitis Optica: Pathogenicity of Patient Immunoglobulin in Vivo. Ann. Neurol..

[167] Geis C., Ritter C., Ruschil C., Weishaupt A., Grunewald B., Stoll G., Holmoy T., Misu T., Fujihara K., Hemmer B. (2015). The Intrinsic Pathogenic Role of Autoantibodies to Aquaporin 4 Mediating Spinal Cord Disease in a Rat Passive-Transfer Model. Exp. Neurol..

[168] Zhang H., Verkman A.S. (2014). Longitudinally Extensive Nmo Spinal Cord Pathology Produced by Passive Transfer of Nmo-Igg in Mice Lacking Complement Inhibitor Cd59. J. Autoimmun..

[169] Matsumoto Y., Kanamori A., Nakamura M., Takahashi T., Nakashima I., Negi A. (2014). Sera from Patients with Seropositive Neuromyelitis Optica Spectral Disorders Caused the Degeneration of Rodent Optic Nerve. Exp. Eye Res..

[170] Redler Y., Levy M. (2020). Rodent Models of Optic Neuritis. Front. Neurol..

[171] Pohl M., Fischer M.-T., Mader S., Schanda K., Kitic M., Sharma R., Wimmer I., Misu T., Fujihara K., Reindl M. (2011). Pathogenic T Cell Responses against Aquaporin 4. Acta Neuropathol..

[172] Jones M.V., Collongues N., De Seze J., Kinoshita M., Nakatsuji Y., Levy M. (2012). Review of Animal Models of Neuromyelitis Optica. Mult. Scler. Relat. Disord..

[173] Ratelade J., Bennett J.L., Verkman A.S. (2011). Intravenous Neuromyelitis Optica Autoantibody in Mice Targets Aquaporin-4 in Peripheral Organs and Area Postrema. PLoS ONE.

[174] Hinson S.R., Roemer S.F., Lucchinetti C.F., Fryer J.P., Kryzer T.J., Chamberlain J.L., Howe C.L., Pittock S.J., Lennon V.A. (2008). Aquaporin-4-Binding Autoantibodies in Patients with Neuromyelitis Optica Impair Glutamate Transport by Down-Regulating Eaat2. J. Exp. Med..

[175] Waters P., Jarius S., Littleton E., Leite M.I., Jacob S., Gray B., Geraldes R., Vale T., Jacob A., Palace J. (2008). Aquaporin-4 Antibodies in Neuromyelitis Optica and Longitudinally Extensive Transverse Myelitis. Arch Neurol..

[176] Oberheim N.A., Wang X., Goldman S., Nedergaard M. (2006). Astrocytic Complexity Distinguishes the Human Brain. Trends Neurosci..

[177] Sanes J.R., Masland R.H. (2015). The Types of Retinal Ganglion Cells: Current Status and Implications for Neuronal Classification. Annu. Rev. Neurosci..

[178] Traub J., Hausser-Kinzel S., Weber M.S. (2020). Differential Effects of Ms Therapeutics on B Cells-Implications for Their Use and Failure in Aqp4-Positive Nmosd Patients. Int. J. Mol. Sci..

[179] Bergman I., Basse P.H., Barmada M.A., Griffin J.A., Cheung N.K. (2000). Comparison of in Vitro Antibody-Targeted Cytotoxicity Using Mouse, Rat and Human Effectors. Cancer Immunol. Immunother..

